# Direct and Indirect Effects of COVID-19 in Frail Elderly: Interventions and Recommendations

**DOI:** 10.3390/jpm11100999

**Published:** 2021-10-02

**Authors:** María Guadalupe Pizano-Escalante, Luis Miguel Anaya-Esparza, Karla Nuño, José de Jesús Rodríguez-Romero, Sughey Gonzalez-Torres, David A. López-de la Mora, Zuamí Villagrán

**Affiliations:** 1 Departamento de Ciencias Biomédicas, Centro Universitario de Tonalá, Universidad de Guadalajara, Nuevo Perif. Ote. 555, Tonalá 45425, Mexico; maria.pizano0484@academicos.udg.mx (M.G.P.-E.); karlajanette.nuno@cutonala.udg.mx (K.N.); 2 Servicio de Geriatría, Hospital Regional Valentín Gómez Farías, Instituto de Seguridad y Servicios Sociales de los Trabajadores del Estado, Av. Soledad Orozco 203, Zapopan 45100, Mexico; 3 Departamento de Ciencias Pecuarias y Agrícolas, Centro Universitario de Los Altos, Universidad de Guadalajara, Av. Rafael Casillas Aceves 1200, Tepatitlán de Morelos 47620, Mexico; luis.aesparza@academicos.udg.mx; 4 Laboratorio Integral de Investigación en Alimentos, Tecnológico Nacional de México—Instituto Tecnológico de Tepic, Av. Tecnológico 2595 Fracc, Lagos del Country, Tepic 63175, Mexico; jrguez344@gmail.com; 5 Departamento de Ciencias de la Salud, Centro Universitario de Los Altos, Universidad de Guadalajara, Av. Rafael Casillas Aceves 1200, Tepatitlán de Morelos 47620, Mexico; sgonzalez@cualtos.udg.mx

**Keywords:** frailty, COVID-19, vulnerability, older people, CGA, functional foods

## Abstract

Frailty is a state of vulnerability to stressors because of a decreased physiological reserve, resulting in poor health outcomes. This state is related to chronic conditions, many of which are risk factors for outcomes in elderly patients having SARS-COV-2. This review aims to describe frailty as a physiological vulnerability agent during the COVID-19 pandemic in elderly patients, summarizing the direct and indirect effects caused by the SARS-COV-2 infection and its prognosis in frail individuals, as well as the interventions and recommendations to reduce their effects. Cohort studies have shown that patients with a Clinical Frailty Scale higher than five have a higher risk of mortality and use of mechanical ventilation after COVID-19; nonetheless, other scales have also associated frailty with longer hospital stays and more severe forms of the disease. Additionally, the indirect effects caused by the pandemic have a negative impact on the health status of older people. Due to the above, a holistic intervention is proposed based on a comprehensive geriatric assessment for frail patients (preventive or post-infection) with emphasis on physical activity and nutritional recommendations, which could be a potential preventive intervention in viral infections by COVID-19.

## 1. Introduction

A novel coronavirus was detected in late 2019 in a seafood market in Wuhan, China, and then was spread worldwide. The etiology of this infection is an acute respiratory syndrome coronavirus 2 (SARS-CoV-2) with a 96% genetic similarity to a bat virus, infecting humans through an intermediary host, rapidly giving rise to a large number of cases [1], so on 11 March 2020, the World Health Organization (WHO) declared the pandemic status caused by COVID-19 [2]. Between March and May 2020, reports in China referred to many cases in people over 60 years and a high fatality associated with comorbidities (diabetes, hypertension, and obesity); it is noteworthy that 69% of the world’s population is older than 60 [3]. Thus, the geriatric population has a higher risk of getting sick and getting a worse prognosis, especially those with a higher age, which could be frailty rather than a chronological age [4,5].

Frailty can be defined as a multidimensional syndrome that reduces the functioning of multiple physiological systems, resulting in the loss of homeostasis and, consequently, the physiological reserve to face internal and external stressors [6,7]. It has been characterized by weight loss, weakness, exhaustion, and low activity [8]. Moreover, there are two main models for its diagnosis, the first one is focused on physical characteristics (phenotype of frailty), and the second is based on the accumulation of deficits according to The Clinical Frailty Scale (CFS) [9]. This scale classifies the frailty severity into nine categories, where advanced categories are associated with a worse prognosis like hospitalizations or death [9], which were more evident in patients with COVID-19 [10]. Independently of the age, frailty has been linked with atypical presentations (functional decline, falls, delirium) [11] and in some cases with an increased in-hospital mortality, days of stay, intensive care unit (ICU) admission, and the need of support with mechanical ventilation [12,13]. This vulnerability is mainly associated with a type 1 interferon (IFN-1) altered response, impaired production of T and B cells, and a secretory phenotype of senescent cells (immunosenescence) [14,15], while considering that older people can be socially vulnerable [16].

Woolford et al. (2020) reported that positive COVID-19 patients were frail and had multimorbidity, independent of the gender and age [17,18]. In this context, diverse authors highlighted the frailty in the pandemic’s clinical results in older patients, associated with the direct (hospitalization, mechanical ventilation, and dead) and indirect (social isolation, malnutrition, physical inactivity) effects and risk factors (i.e., depression, anxiety, sarcopenia, obesity, commorbidities, and hospitalization) [17,18,19]. In this context, the CFS > 6 has been linked to an increase in mortality risks, independently of age and gender [19]. Even a higher risk of mechanical ventilation was predicted after adjusting age, gender, and comorbidity with higher CFS scores, while lower CFS scores can be interpreted as protective and earlier discharge [20].

This narrative review aims to describe frailty as a physiological vulnerability agent during the COVID-19 pandemic. It summarizes the direct (hospitalization or death) and indirect (physical, social, and psychological) effects caused by COVID-19 infection and its prognosis in frail individuals, as well as the interventions and recommendations to reduce their effects.

## 2. Materials and Methods

### Sources of the Data and Search Strategy

This study aimed to review the available reports on the direct and indirect effects of the COVID-19 pandemic in frail persons. For this, a comprehensive search was performed online through the PubMed and SCOPUS databases using the search pattern TITLE-ABS-KEY (“Frailty AND COVID-19”) OR TITLE-ABS-KEY (“Frailty AND Coronavirus) OR TITLE-ABS-KEY (“Frailty AND Novel coronavirus infection). The period of publication was from 2020 to 2021. However, publications from recent years were considered to the review as a theoretical framework of frailty and its diagnosis. In the present narrative review, all retrieved publications that met the inclusion criteria were considered (meta-analysis, bibliometric analysis, and editor letters).

Additionally, bibliometric maps were performed to visualize and analyze trends of frailty and COVID-19. In this context, a stratified search was carried out using the Carrot2 software (clustering algorithm Lingo) and data obtained from the PubMed database using the search pattern “Frailty” AND “COVID-19” as a guide to identify relevant information about frailty and COVID-19. Furthermore, VOSviewer software (version 1.6.16) was used to analyze the distribution and connection of searching terms on recently published papers about Frailty and COVID-19. The results were based on the threshold of 252 terms from 3672 keywords from 612 documents focused in four clusters, where each node or circle in the map represents a term at least ten times, and the node or circle is proportional to the number of occurrences of that term. Data was obtained from the SCOPUS database using the search pattern TITLE-ABS-KEY: Frailty AND COVID-19.

## 3. Frailty: A Vulnerability State

Aging is one of the main factors for a vulnerable state of life due to the decline of physiological functions and a determining factor for pathological aging [13]. WHO defines frailty as a “*progressive age-related decline of body functions resulting in vulnerability and reduced resilience to physical and mental stressors with an increased risk of negative health outcomes*” [21]. Furthermore, frailty has been described as a deficit accumulation in many physiological systems [20]. Currently, there is not a gold standard to determine frailty, and many tools need to be used to diagnose this condition. We need a broader clinical picture than the simple chronological age [22]. Table 1 lists the clinical characteristics of frail patients.

The theoretical concept of decline of function and disability associated with age appeared among geriatricians three decades ago, but the attention in the scientific literature arose in the 90s and snowballed with the work of Fried et al. [8] in 2001.

To date, there are more than 40 ways to assess frailty without a consensus on the most appropriate method [23,24]. The original operationalization of the frailty phenotype by Fried et al. [8] was described based on a cohort from a cardiovascular study. They describe a frailty circle of interrelated physical manifestations culminating in a vulnerability state characterized by weight loss, weakness, self-report of exhaustion, slowness, and low physical activity. These characteristics are related to a higher risk of falls, worsening mobility, disability, hospitalization, and death within the next three years, constituting the most popular tools for the diagnosis [8].

Rockwood et al. [9] created a model of the disease based on the accumulation of clinical deficits, named the Frailty Index (FI). This model considers the damage through molecules, cells, organs, and systems that eventually manifest as frailty [25,26]. Later, derived from this index, they described the CFS, a more practical tool in the clinic [23].

The CFS classifies the patients on a nine-degree scale in agreement to severity, 1–4 are patients without frailty (robust or fit), 5 is mildly frail, 6 is moderately frail and, 7–9 are patients who are severely frail. A meta-analysis reported an increase of 12% in mortality for each progression point in the CFS [27]. Therefore, the National Institute for Health and Care Excellence (NICE) recommended its use for decision-making during the COVID-19 pandemic [27,28]. Moreover, another valuable tool for frailty diagnosis is the “Hospital Frailty Risk Score” (HFRS) developed and validated in 2018 by Gilbert et al. [29]. This score uses the International Classification of Disease-10-Clinical Modification (ICD-10-CM) diagnosis codes to calculate the risk of frailty in three groups: low (<5 points), intermediate (5–15 points), and high (>15 points) [12].

Beyond clinical classification, frailty is a vulnerable state that impacts the physiological reserve required to face acute stressors leading the body to deteriorate, resulting in a poor clinical prognosis [22], strongly impacted by COVID-19.

## 4. Frailty and COVID-19

**Figure 1:**
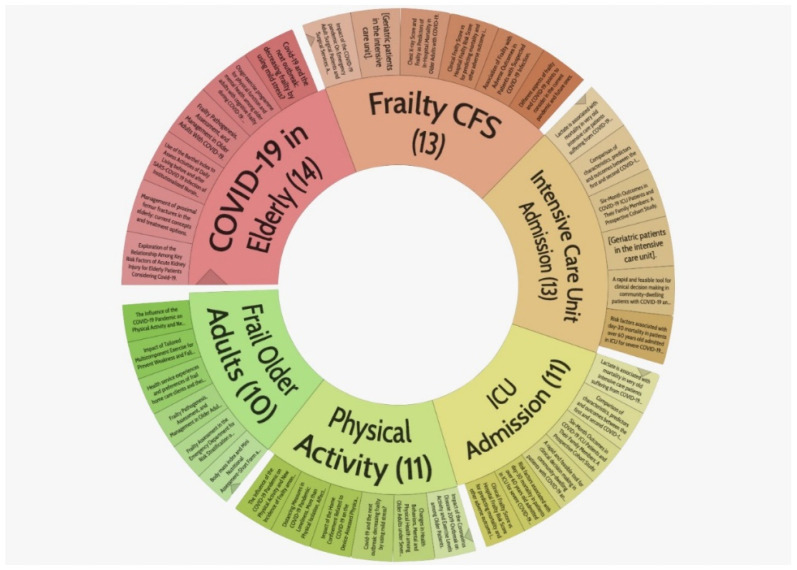
Stratified search clustering COVID-19 infections in geriatric elderly patients and its relationship with frailty and its severity evaluated by the Clinical Frailty Scale. The figure was created using Carrot2 software by clustering algorithm Lingo and data obtained from the PubMed database using the search pattern “Frailty” AND “COVID-19” and all retrieved results. Available in https://search.carrot2.org/#/search/pubmed/frailty%20and%20COVID-19/pie-chart (Accessed on 22 August 2021).

Frailty and COVID-19 have been associated with a negative effect on older people [5]. The evidence about the relationship between frailty and COVID-19 infection was estimated using a stratified search to understand how frailty and comorbidities in elderly patients have influenced the presentation and prognosis of the disease. Figure 1 describes the current interest in the search for interactions between COVID-19 and the elderly, specifically those with frailty, and the adverse health outcomes intrinsic to this condition.

Many authors have described the characteristics of the elderly population with the COVID-19 infection in diverse cohorts, particularly the strong relationship between comorbidities and frailty status related to deleterious results (Table 2). Steinmeyer et al. [30] describes a population with a mean age of 85 years, most living in a community, and reports 69% had hypertension, 48% had cardiac disease, 45% had dementia, 37% had respiratory disease, and 11% had diabetes mellitus. In addition, many geriatric syndromes were present, for example, polypharmacy (44–65%), need for mobility aids (30%), cognitive impairment (19.4%), institutionalization (10%), and frailty syndrome (10%). The atypical presentation of the disease is frequent among the elderly (48% of falls, delirium, and malaise) and the length of stay was 12 days in most patients [30,31]. Table 2 describes the characteristics of frail geriatrics patients with COVID-19 and their related morbidities.

**Figure 2:**
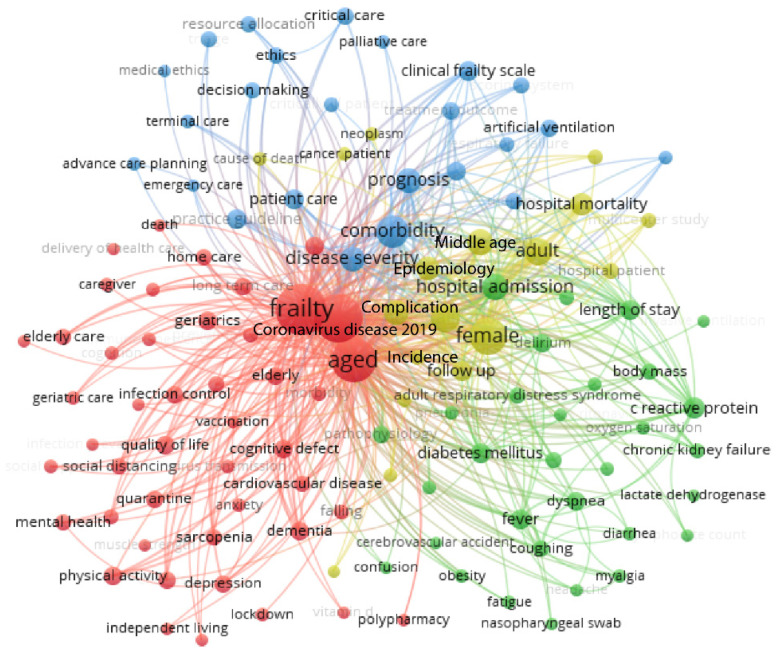
Distribution of searching terms on recently published papers about Frailty and COVID-19. Figure created with VOSviewer version 1.6.16 software. The results are based on the threshold of 252 terms (from 3672 keywords) with four clusters, where each node or circle in the map represent a term at least 10 times and the node or circle is proportional to the number of occurrences of that term, data obtained from SCOPUS database using the search pattern TITLE-ABS-KEY: Frailty AND COVID-19 (Accessed on 22 May 2021).

According to the evidence, frail patients can survive a COVID-19 infection with a slight association between this condition and the increase in mortality [13]. Figure 2 shows the distribution of terms recently published (2020-2021 from the SCOPUS database) in scientific articles related to COVID-19 infections in frail elderly patients. It can be observed that this distribution is centered in four clusters where the first one (red color) includes aging, frailty, and its influence on COVID-19 infections. The second cluster (yellow color) refers to the epidemiology of the disease, revealing an association between its incidence and hospital admission with a poor prognosis. On the other hand, the third cluster (blue color) describes ethical aspects like mechanical ventilation or admission to intensive care units, highlighting the use of the clinical frailty scale. At the same time, the fourth cluster (green color) mentions the most important clinical manifestations and comorbidities. Therefore, it is important to classify the COVID-19 disease effects described in frailty patients as direct and indirect, as discussed below.

### 4.1. Direct Effects of COVID-19 in Frail Population

From the beginning of the pandemic in 2019, research groups have shown a great interest in COVID-19 and its effects on the elderly, particularly in frail elderly (Table 3).

The direct effects of a COVID-19 infection in older people are mainly determined by a state of immune dysfunction. These effects are characterized by altered homeostasis of cytokines with an abnormal release of interleukin 6 (IL6), tumor necrosis factor α (TNF-α), C-reactive protein (PCR), and diminution of anti-inflammatory substances like interleukin 10 (IL10) with a decrease in the number of B and T cells as well as the response to IFN, essential for the antigen response. Consequently, to stop the progression of viral infections, especially to new antigens, this state is named “immunosenescence”; it contributes to many age-related chronic diseases [14,15,36,37].

In general, these immunological modifications contribute to the poor prognosis in the elderly since severe cases have been associated with elevated levels of inflammatory cytokines such as IL6, TNF-α, and interferon-γ (IFN-γ), as well as lower lymphocyte counts (specifically CD8+ and CD4+) [15], mainly attributable to an inadequate immune response, proinflammatory basal state and consequent inability to overcome the inflammatory process associated with viral pneumonia and tissue injury leading to acute respiratory distress syndrome (ARDS) [18]. Moreover, other mechanisms have been linked to the predisposition of older people to develop a severe disease by a COVID-19 infection. For example, the modifications in the expression of the human angiotensin-converting enzyme II (ACE2) (receptor used by the virus for entering the cell) attributed to the age and, although not consistently, related to the pathogen lethality [15,37]. Furthermore, another possibly related process is the increase of reactive oxygen species (ROS) (due to poor clearance in an older person), which causes hyperstimulation that may increase the inflammatory state [38].

In the United States, one of the countries with the highest number of infected patients worldwide, 31–59% of hospitalizations have been for patients over 65 years, and death increased dramatically above the 80 years threshold, with a case fatality rate of 21.9%. Moreover, it was reported that there is an increased mortality risk associated with age and CFS > 5 compared with survivors (IQR 4–6 vs. 3.5, IQR 2–5; *p* < 0.01), as well as greater opportunities of hospital discharge with a CFS < 5 [31].

The intrinsic effects of aging have been mainly related to the prognosis, proving to be much worse in frailty patients than in older fit patients. Although with variable results, CSF has been reported in many cohorts as a useful tool for frailty detection and risk classification. An international, multicenter cohort (including 63 hospitals in Europe) reported an increased risk of hospital mortality in 65 years old frail patients (CFS 6–9) compared with fit ones (CFS 1-3) without more risks of admission to intensive care, the last attributed to mayor comorbidities that may predispose the medical team not to consider them as candidates [39]. Other single-center studies describe a relationship between CFS categories and mortality, R. De Smet et al. [27] demonstrated an elevated death rate in the CFS categories > 7, being the risk greater from a category higher than 6 in other reports [35].

In the evaluation of Haagg et al. [21], a CFS > 5 was correlated with a higher death risk and lowered expectations of discharge, with a 24% global mortality of the frail population in this study. Likewise, no matter the frailty degree (mild, moderate or, severe), using the CFS in a metanalysis, it was found an elevated hazard ratio of mortality associated with frailty status [40], and even the FI > 0.25 compared to < 0.25 correlates with risk mortality and ICU admission [41]. As described by Tehrani et al. [33], the frailty status has resulted in a better independent predictor of death than age and comorbidities. Even though there is a strong relationship between the CFS and mortality, especially with a superior level of five, not all the results are homogeneous. Owen et al. [42] found a relationship with mortality only in the group of CFS 9 (in the adjusted analysis) without an increase in mortality by increasing the CFS scores. Chinnadurai et al. in 2020 showed an association (using a multivariate statistical tool) between infected frailty patients (CSF > 5) with COVID-19 and sociodemographic characteristics, where mortality of the infected increased about 40% [43]. On the other hand, Steinmeyer et al. [30] did not find an association between CFS and the described factors. The HFRS has also been used to determine the prognosis of frail patients, and demonstrated an association with mortality, prolonged hospitalization, and the need for mechanical ventilation [12].

For patients in long care facilities, frailty is a highly prevalent condition, and the results associated with a COVID-19 infection are equally severe, and the number of deaths have been over 72% [35]. Labenz et al. [20], in a retrospective cohort, describes the main use of mechanical ventilation in patients with higher CFS scores and a reduction in the days of hospitalization (univariable COX regression analyzes). The progression to a severe disease is influenced similarly by the frailty status [18].

According to the evidence, there is a relationship between the severity of frailty and the development of a severe COVID-19 condition with and without hospital admission. In this context, frailty is a high-risk factor independent of multimorbidity, lifestyle, and sociodemographic factors regardless of the frailty model [34].

### 4.2. Indirect Effects of COVID-19 in Frail Population

**Figure 3:**
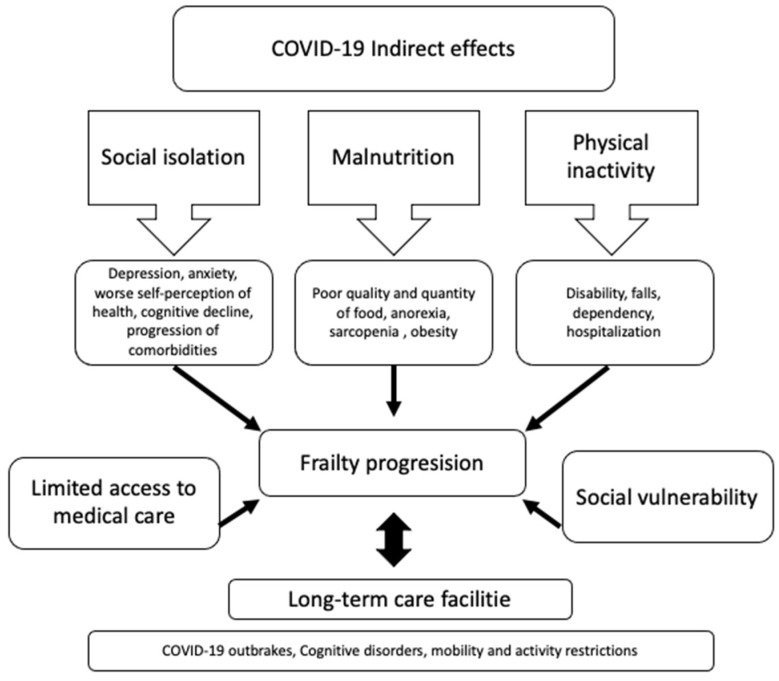
Indirect effects associated with COVID-19 infection leading to progression of frailty.

The most important indirect effects in the elder population caused during the COVID-19 pandemic are related to social isolation, as many people had to stop their lifestyle and assume a voluntary quarantine with its consequences. Figure 3 shows the interactions between the main indirect effects (social isolation, malnutrition, physical inactivity) and their association with some risk factors (i.e., depression, anxiety, sarcopenia, obesity, commorbidities, and hospitalization) that determine a progression of the frailty condition (pre-pandemic or post-pandemic).

Recommendations of isolation for the elderly caused a locked up in their homes, in many occasions without recreational or work activities and the interaction of friends or even their own families being considered as non-core activities [44,45]. Even in normal circumstances, older people tend to be more isolated than the younger ones [46]; in this sense, the adverse effects appear to be worse for people with disability, multimorbidity, and frailty [47]. These consequences include the perception of loneliness, an intensified depression, anxiety, worse self-perception of physical health, cognitive decline, and the risk of cardiovascular disease, obesity, and stroke [44,46,47]. Additionally, social isolation represents a risk factor for frailty progression [48].

Additionally, psychiatric conditions like depression and anxiety disorders are common among the geriatric population, with a prevalence of 1.2 to 15% [49], which were increased during the pandemic due to health concerns and the risk of contagion [49]. Brooke et al. [50], through a qualitative study (with great insights), describes how older people perceive social distancing and acknowledge the need to follow the restrictions as well as the constant concern about the risk they take by getting the infection. It has been proven that this population group consistently expresses negative feelings related to stress, anxiety, and concern about COVID-19 [47].

Patients in long-term care units have suffered due to their vulnerable condition, and the high mortality in this group has led to the need for stricter social restrictions, which at least in Europe has reached 79% [44,51]. In these populations, the progression of frailty has been dramatic because of the sum of factors like isolation of family members, pre-existing cognitive disorders, limitations on regular medication, and even the presence of COVID-19 outbreaks in the same institutions. Around the world, outbreaks have placed them as epicenters of the pandemic because of several reasons, including individual, community, institutional and contextual policy levels [51].

The impact of the indirect effects of the disease can be seen in multiple health conditions, like nutrition, causing an inability to access appropriate food items in terms of quality and quantity, or the risk to develop anorexia, among others. Therefore, the nutritional status must be a focal point due to the relationship between obesity and poor outcomes associated with COVID-19 infection, and the consequences of malnutrition in the immune system, especially in frailty sarcopenic patients that negatively influenced the time of recovery of the disease, the mobility and respiratory function [52,53]. Accordingly, the screening of the nutritional state may contribute to the evaluation of the elderly.

Likewise, mobility disorders represent one of the main pathological conditions in the elderly; in addition to the aforementioned factors, the opportunities for adequate physical activity have been reduced. Consequently, inactivity and its consequences represent a major effect of the current pandemic. This does not only mean that those who used to do continuous physical activity stop doing it, but also that elderly with previous mobility impairments developed more their disability, falls, need for assistance, hospitalization, and finally a worse health status [54,55].

In the worst of the pandemic, some countries (especially low and middle-income countries populations, who represent 69% of the total people over 60 years old) had to make decisions about resource allocations based on the biological age, which is ethically and legally debatable, besides the enormous difficulties in maintaining adequate recommended measures to prevent contagion due to their family systems (with the constant and obligate interactions with the youngest), access to health, living conditions and no regulated long-term care units [3,16]. This situation can occur, especially, in some areas where the required number of intensive care unit beds, mechanical ventilation, or medical personnel are being exceeded. Therefore, the contribution of frailty to the individual prognosis could be an enormous contribution to decision-making [56].

In this extraordinary moment, the world needs strategies and specific guidelines to minimize the indirect effects of COVID-19 in elderly patients and the general population.

## 5. Interventions to Reduce Frailty

Frailty is described as a dynamic state in which patients may progress rapidly due to acute illness; however, some interventions could reduce its severity [57]. In this sense, in patients who have undergone COVID-19 and in those with high vulnerability due to indirect effects, various interventions should take place as part of a comprehensive treatment plan to reduce frailty and even prevent this condition in healthy elder people.

Although clinical studies have not consistently demonstrated its effectiveness in reducing frailty, the Comprehensive Geriatric Assessment (CGA) is considered a fundamental tool in its evaluation [58,59]. The initial approach aims to create an individualized, patient-centered, and ideally a multidisciplinary intervention plan including the work of the geriatrician, physiotherapist, nutritionist, social worker, psychologist, patient, and caregivers [57,60]. The CGA allows the identification of comorbidities and pharmacology therapy to identify the presence of polypharmacy and signs and symptoms like exhaustion and weight loss (first manifestation of frailty) [48,59]. Because of these highly nonspecific initial symptoms of frailty, a search using clinical scales, according to the model of physical phenotype or accumulation of deficits, allows for early interventions to reduce or reverse this clinical condition.

**Figure 4:**
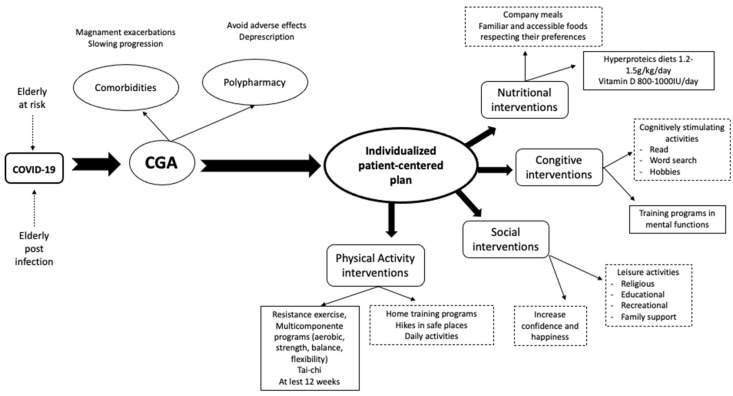
Proposal diagram for intervention in the frail patient after COVID-19 infection or at risk of infection. CGA (Comprehensive Geriatric Assessment).

A proposed diagram for intervention in the frail patient after a COVID-19 infection or at risk of infection, covering four main areas: cognitive, nutritional, physical, and social, is shown in Figure 4. The interventions that integrate the individualized person-centered plan integrated for treatment of chronic diseases to prevent their progression and control their symptoms, could be a viable strategy to reduce frailty.

### 5.1. Evidence-Based Recommendations

It has been previously reported in patients with COVID-19 that social isolation may independently increase the risk of physical frailty four years later [61]. Although evidence has not demonstrated the existence of social frailty, strengthening social networks can reduce levels of the disease by improving independence, maintaining a healthier diet, more frequent physical activity, and curbing excessive consumption of alcohol or tobacco [62]. Specific social leisure activities have been evaluated (social clubs and programs, volunteer activities, religious activities, education programs, visit art exhibitions, theater, or cinema) and demonstrated their effect on the relationship between the frequency of the activities and the reduction in the progression of frailty [63,64].

For the cognitive sphere, the evidence suggests the benefit of specific programs, Ng et al. [65] evaluated the effect of cognitive training with activities focused on stimulating short memory, attention, and information-processing skills, finding a reduction in frail scores after 12 months of tracking alone or in combination with exercise and nutritional management. Lastly, maintaining a healthy diet and regular physical activity is more beneficial for good mental health; although specific supplements with antioxidant effects (vitamins, minerals, fatty acids) have been tested, results have not been conclusive [66]. Despite the above, there is evidence that healthy eating styles can be beneficial, the Mediterranean and DASH (dietary approaches to stop hypertension) diets based on fruits, vegetables, legumes, limiting the consumption of red meat and saturated fats, reduce cognitive decline and the risk of dementia [66].

There is an interest in vitamin D because of its association with better physical performance and now emerges as a promising target. Annweiler et al. [67], in a quasi-experimental study, found a reduction in severity and mortality at 14 days in a group with the previous exposition to the vitamin at least for a year by comparing with a group without the supplementation. Other micronutrients have been associated with reducing the prevalence of the syndrome (α-tocopherol, vitamin C, B6, A, β-carotene, folate) [68]. For the macronutrients, protein is the more relevant, regardless of the source; meals have to contain high-quality protein considering that the meal should be weighted [69]. Although a healthy diet proves to be beneficial, like the Mediterranean diet, which has decreased the risk of frailty, it is known that an intake of less than 1g kg^−^^1^ of protein affects the strength and physical performance [70].

Although the necessary protein intake in patients with frailty has not been established, and it seems that this amount may vary according to our treatment purpose, the quality is important, finding that higher amounts of leucine activate protein synthesis [71]. In a randomized control trial with 12 weeks of intervention, Park et al. [71] found that an intake of 1.5 g per kg of weight improves the appendicular skeletal muscle mass in weight, height, and body mass index, improving physical performance as assessed by gait speed compared with the ingestion of 0.8–1.2 g/kg/day. Even with the consumption of amounts of protein that might seem sufficient, evidence has shown the need to encourage physical activity.

In frail patients, the concurrence of sarcopenia is high; in these circumstances, physical exercise is indispensable. The latter tries hypertrophy in the fibers while the synthesis is stimulated with the intake of amino acids [72]. Physical activity has been shown to reduce the risk and reverse the state of frailty, improving the cognitive and affective state that positively influenced the life quality, balance, and mobility. It is recommended for interventions of at least 12 weeks with sessions of 30 to 60 min either with strength or multicomponent exercises; the latter being very useful in patients with low cardiovascular capacity since it involves balance activities, aerobic exercise and even functional activity [73,74,75].

Despite the above evidence, the application of both interventions (simultaneously) proves to be the most effective [76]. A meta-analysis reported that the combination of physical exercise (including resistance and multicomponent) and nutritional interventions with protein supplementation (ranging from 3 to 40 g day^−^^1^ for 12 to 24 weeks) promoted an increase in muscle mass with a positive effect on leg strength which translated into a significant effect on gait [73].

In general, applying all these interventions (multidomain interventions) could be the most beneficial to holistically treat elderly patients, improving their frailty status and reducing the direct and indirect effects promoted by COVID-19.

#### Functional Foods

According to the Functional Food Center, functional foods (FF) are “Natural or processed foods that contain biologically-active compounds which are effective in non-toxic amounts, providing a clinically proven and documented health benefit utilizing specific biomarkers, for improving general health, for the prevention, management, and treatment of chronic and viral disease or its symptoms” [77]. Their benefits occur with amounts normally consumed in the diet; these can be food ingredients or whole foods that include micronutrients, probiotics, flavonoids and carotenoids, as well as herbs with potential beneficial effects in the immune system independent of nutritional ones [78].

Although the main application of FF has focused on non-communicable diseases, recently, it was proposed that FF could prevent the risk of contagion or fight infectious diseases such as COVID-19, based on the fact that most of the patients affected by this infection suffer from these conditions, in addition to a deficient immune system, both of which are associated with aging [77,79]. Generally, the potential effect is secondary to the immune-boosting properties reducing the stress levels, and in consequence, these compounds may play an essential role in preventing infectious diseases, like the COVID-19, not actually by inhibiting the virus directly. However, for its preventive effects, in this sense, they maintain the gut health through the preservation of the intestinal microbiota and its relationship with an adequate immune response [78,80].

The following describes some of the demonstrated and potential effects of various FFs and their health benefits in patients with COVID-19.

Vitamin A and B contained in various fruits and vegetables, have beneficial effects on the immune function, such as modulation of antibody formation, reduction in the levels of inflammatory cytokines, stimulation of both innate and adaptive responses (maturation, proliferation, and improved functions of various cell groups such as lymphocytes, neutrophils, and natural killers), and their antioxidant and oxidative stress reduction effects [81,82]. Likewise, vitamin C (present in cherry, oranges, lemon, broccoli, and peppers) is recognized for its protective effect against viral diseases such as the common cold; this depends on its antioxidant, anti-inflammatory and cell-specific effects such as leukocyte stimulation (both for migration and antibody production) and natural killer stimulation, so its consumption from foods could be a good alternative to reduce the risk of COVID-19 contagion [82]. Furthermore, the consumption of food rich in vitamin D (eggs, tuna, salmon, and dairy products) may be helpful against the so-called “cytokine storm” due to its reduction of IL6, interleukin-8 (IL-8), interleukin-12 (IL-12), TNF-α, and IFN-γ production [83]. Similarly, food rich in vitamin E (nuts, vegetable oils, and wheat germ) has potential to reduce COVID-19 contagion due to their antioxidant properties [82].

Minerals such as zinc, selenium, iron, and other trace minerals present in large quantities in seeds and nuts also have an important antioxidant and anti-inflammatory effects by increasing the cytotoxic action of lymphocytes and natural killers [78]. Likewise, lipids such as omega-3 fatty acids, eicosapentaenoic acid, and docosahexaenoic acid mainly present in fish and seafood could improve the inflammatory state through an adequate immune response [78].

The group of polyphenols (flavonoids and phenolic acids) that can be found in fruits and vegetables can interfere with the release of certain cytokines and therefore modify innate and adaptive immunity (which provide an important anti-inflammatory effect) as well as possessing antioxidant, antitumor, antibacterial, and antiviral properties [84,85,86]. Resveratrol can be found mainly in grapes, berries, mulberry, and peanuts and has demonstrated by in vitro studies an effect against respiratory viruses including SARS-CoV, probably by the inhibition of viral ribonucleic acid (RNA) replication [82,85]. Moreover, there are also foods with potential antiviral effects, especially those rich in caffeic acid (commonly found in fruits, vegetables, chocolate, and coffee) that have this potential effect. In silico studies have shown inhibition of the interaction of the COVID-19 virus with the angiotensin-converting enzyme receptor 2 (ACE2), whose proteins are inhibited by organosulfur compounds contained in garlic essential oil [80,87]. In addition to the flavonoid group, alkaloids and terpenes decrease the production of inflammatory cytokines and chemokines (especially interleukin-1 (IL1), IL6, and TNF α), which reduces oxidative stress and thus the inflammatory processes [78,86].

Similarly, some traditional herbs and roots (*Cinnamomic cortex*, *Armeniacae semen*, and *Ephedrae herba*) have a potential antiviral effect by decreasing the viral performance and stimulating the immunoglobulin production [80]. One of the most popular beverages is green tea (*Camellia sinensis*), included in this group, which possesses an antiviral effect that improves the innate immunity providing anti-inflammatory effects [88].

Additionally, it has been reported that the intestines are often affected during COVID-19 with diarrhea symptoms; therefore, consuming foods rich in probiotics (e.g., yogurt) is an alternative to improve intestinal function [82].

It should be noted that most of the information related to functional foods and COVID-19 are review in meta-analysis articles, in silico studies or letters to the editor, in which, based on scientific evidence, their consumption is proposed as an alternative to modulate the immune system of people with a positive impact on the prevention and treatment of COVID-19, particularly in older people.

### 5.2. Experience-Based Interventions

The absence of interventions with a high degree of recommendation should not lead to no intervention at all, especially through low-cost actions. The following are interventions that may be of benefit according to the author’s experience.

For cognitive stimulation, continuous interaction is essential; an environment that allows them to intervene in schedule activities at home, forcing them to know the dates and times of the activities. Moreover, everyday conversations, whether in person, on the telephone, or other electronic media, can stimulate memory about recent or past events. Finally, it is appropriate to advise activities such as recreational reading, like the newspaper, word search puzzles, and even handicrafts (e.g., embroidery for those who are used to them).

Integration into pleasant activities can increase confidence and happiness; although during the pandemic, the possibilities may be restricted, the nuclear family can be the source of this well-being participation in activities within the home such as family meals, board games, or reminiscences. Even intergenerational coexistence can generate a feeling of integration in older people, giving security and support from his or her family. In this sense, appropriate family support may positively influence the health status of the elderly.

Eating is considered as a social act, but patients who live alone, or eat alone, often skip meals or eat in small quantities. Healthy food with the inclusion of all nutrient groups may be the general recommendation. However, it should take into consideration the individual’s food access, preferences, and economic situation. Despite the above, it is important to insist on the intake of protein, whether of animal or vegetable origin, at every mealtime and avoid substituting the diet with commercial supplements that usually end up being the patient’s only food when used continuously.

Finally, physical activity will depend on the severity of frailty. The logical steps of sitting up, getting out of bed, standing, and walking must be accomplished before training programs can be attempted. The main issue is to achieve continuous mobility, either in or out of bed, using technological tools (videos, applications) to start training programs adapted to the patient’s physical situation. We recommend using the home-based programs of Vivifrail excercise for elderly adult programs available on the page https://vivifrail.com/resources/ (accessed on 8 June 2021). Even if the person is not used to exercise or is not motivated to do these kinds of programs, we can recommend activities such as walking in safe places, swimming, cycling and even continue with activities at home, from housework to gardening.

According to the experience-based recommendations, continuous interaction of frailty patients with their families improves their health status. Likewise, it is necessary to create public policies that promote and facilitate the support and care of older people, particularly frail persons.

## 6. Concluding Remarks

Frailty refers to a state of vulnerability to stressors characterized by a decreased physiological reserve, resulting in poor health outcomes. According to the revised literature, reports about the direct and indirect effects promoted by COVID-19 in frailty are scarce. Moreover, these documents include meta-analysis, bibliometric analysis, and editor letters, which is the main limitation during the pandemic situation that may increase the vulnerable status of frail persons by an inadequate intervention by health professionals.

Additionally, there is no standardized methodology to determine who is frail. Nonetheless, this vulnerable state has increased in older people by the COVID-19 pandemic effects (direct or indirect), becoming a high-risk factor to develop a severe infection of this respiratory disease. The main direct effects include hospitalization, admission to intensive unit care, and death. Contrary to the indirect effects: social isolation, malnutrition, and physical inactivity that may promote depression, anxiety, sarcopenia, and hospitalization that together may exhibit a progressive effect on the frailty status (pre-pandemic or post-pandemic).

It is imperative to establish multidisciplinary interventions that help to reduce the adverse effects in frail people with and without COVID-19 infection. These interventions include cognitive stimulation, integration into pleasure activities, healthy eating habits, and physical activities, which are low-cost and easy actions that may improve the quality of life of elderly patients.

On the other hand, it is necessary to broadcast information about the characteristics and consequences of frailty as a disease to facilitate its early diagnosis among the general population and the medical community. Moreover, it is also essential to invest in public policies focused on the healthcare of frail people and promote among health professionals the importance of attending to the needs of patients and those of their families. However, further studies are still required to standardize protocols for the diagnosis and treatment of frailty.

## Figures and Tables

**Table 1:** Identification of clinical characteristics of frail patients by model disease.

Model	Clinical Characteristics	References
Physical phenotype	Weight loss and Sarcopenia Weakness Exhaustation self-report Slow walking speed Low physical activity	Fried et al. [8]
Accumulation of clinical deficits	Detrimental to health conditions Age-related Failure of multiple physiological systems	Rockwood [9]

Adapted from Fried (2001) and Searle (2008).

**Table 2:** Characteristics of geriatric patients with COVID-19 and morbidities associated.

Study Type	Frailty Prevalence (%)	Average Age (Years)	Residential Status	Comorbidities	Geriatric Syndromes	Ref.
Retrospective cohort	10%	85	Community	Hypertension 69.1%, Cardiac disease 48.9%, Dementia 45.7%, Respiratory disease 37.2%, Diabetes mellitus 11.7%	ADL dependency 64.9%, IADL dependency 76.1%, Polypharmacy 69.1%, Malnutrition 44.7%	[30]
Cross-sectional	14%	81	NI	Hypertension 58%, Diabetes mellitus 31%, COPD 19%, Dementia 15%, Chronic kidney disease 14%	NI	[21]
Cross-sectional	50% markers frailty	68.7	Community and home residents	Hypertension 45%, obesity 31%, diabetes 23%, dementia 15%	Polypharmacy 30%, Mobility aids 10%, Cognitive impairment 19.4%, Delirium 21%, Falls 8%	[31]
Cross-sectional	67.4% (HFRS>5)	74.1	Community	Hypertension 78.8%, CAD 39.3%, Diabetes mellitus 36.2%, COPD 35.9%, Iron deficiency anemia 27.2%, Cerebrovascular disease 18.9%, Renal failure 8.8%, Depression 21.8%, Cancer 7.8%.	NI	[12]
Cross-sectional	66.9% (CFS > 5)	79.9	Community	Diabetes 28%, CAD 26.9%, Hypertension 56.1%, COPD 14.5%, Heart failure 12.6%	NI	[32]
Retrospective, observational	ND	59	Community	Diabetes mellitus 22.5% Hypertension 38.1% Hyperlipidaemia 44.7% CAD 12.3% CKD 2.9% COPD 6.1%	Polypharmacy 26.5% Chronic pain 7.6% Memory problems 2.5% Nutritional risk 6.9%	[13]
Retrospective cohort	74%	66	Community	Hypertension 54% Diabetes 31% CKD 19% CAD 13% Stroke 9% COPD 5% Dementia 6% Cancer 5%	NI	[33]

Markers frailty: falls, polypharmacy, cognitive impairment, dementia, mobility aids, package of care, care home resident. Activity of daily living (ADL), Instrumental activity of daily living (IADL), Coronary artery disease (CAD), Clinical frailty scale (CFS), Chronic obstructive pulmonary disease (COPD), Chronic kidney disease (CKD), Hospital frailty risk score (HFRS). NI (no information).

**Table 3:** Direct efects of COVID-19 in frailty patients.

Frailty Model	Age (Years)	Participants	Category	Clinical Outcome	Study Design	Ref.
Fried phenotype FI	37–73	802	Frail and pre-frail	Increased in severity of disease for both models	Cohort multicentric	[34]
CFS	65–97	81	CFS > 7	No survivors were frailer	Restrospective, single-center observational	[27]
CSF	82–91	289 in hospital 341 in nursing homes	CFS >6	Significantly associated with mortality after 30 days	Retrospective, observational, longitudinal	[35]
Frail Non-Disabled survey	62–99	94	Frail	No correlated with mortality	Retrospective cohort study	[30]
CFS HFRS	Median age 81	967 (250 patients with COVID-19)	CFS > 5	Associated with in-hospital mortality and decreased probability of being discharge. No HFRS relationship found	Cross-sectional single center	[21]
CFS	54–72	42	Higher CFS scores CFS < 3	Higher risk of mechanical ventilation. Correlated with earlier and more frequently discharge from home	Retrospective cohort study, single center	[20]
HFRS	Mean age 74.1	18,234	>5 points	Correlated with all-cause in-hospital mortality, long stay (more than ten days) and use of mechanical ventilation	Cross-sectional, multicenter	[12]
FRAIL	60–96	114	Frail vs. no frail	Association with severe disease	Prospective cohort study	[18]

CFS (clinical frailty scale), FI (frailty index), HFRS (Hospital frailty risk score).

## Data Availability

Not applicable.
